# Stathmin involvement in the maternal embryonic leucine zipper kinase pathway in glioblastoma

**DOI:** 10.1186/s12953-016-0094-9

**Published:** 2016-03-11

**Authors:** Suely Kazue Nagahashi Marie, Sueli Mieko Oba-Shinjo, Roseli da Silva, Marcela Gimenez, Gisele Nunes Reis, Jean-Pierre Tassan, Jose Cesar Rosa, Miyuki Uno

**Affiliations:** Laboratory of Molecular and Cellular Biology (LIM 15), Department of Neurology, School of Medicine, University of São Paul, Av. Dr Arnaldo 455, Cerqueira César, São Paulo, SP 01246-903 Brazil; Center for Studies of Cellular and Molecular Therapy (NETCEM), University of Sao Paulo, São Paulo, Brazil; Protein Chemistry Center and Department of Molecular and Cell Biology, Medical School of Ribeirão Preto, University of São Paulo, Av. Bandeirantes, 3900, Ribeirão Preto, 14049-900 Brazil; Cell Cycle Group, SFR Biosit, UMR 6290 CNRS Institut de Génétique et Développement de Rennes–Université de Rennes 1, 2 Avenue du Professeur Léon Bernard, CS 34317, 35043 Rennes, Bretagne France; Center of Translational Research in Oncology, Instituto do Câncer do Estado de São Paulo-ICESP, Av. Dr Arnaldo 251, 8th floor, Cerqueira César, São Paulo, SP 01246-000 Brazil

**Keywords:** Astrocytomas, MELK, Proteomics, qRT-PCR, Stathmin

## Abstract

**Background:**

Maternal Embryonic Leucine Zipper Kinase (MELK) is a serine/threonine kinase involved in cell cycle, differentiation, proliferation, and apoptosis. These multiple features are consistent with it being a potential anticancer target. Nevertheless, the MELK pathway in tumorigenesis is not yet completely understood. This study aims to identify proteins associated with MELK pathway in astrocytomas. To this end, proteomic data of the human glioma cell line U87MG transfected with siRNA for *MELK* were compared with non-target transfected control cells and compared with oligonucleotide microarray data.

**Results:**

In both assays, we identified stathmin/oncoprotein 18 (STMN1), involved in cell cycle. *STMN1* gene expression was further assessed in a series of 154 astrocytomas and 22 non-neoplastic brain samples by qRT-PCR. *STMN1* expression was significantly increased in malignant diffusely infiltrative astrocytomas compared with pilocytic astrocytoma (*p* < 0.0001). A strong correlation between *MELK* and *STMN1* expressions was observed (*r* = 0.741, *p* < 0.0001) in glioblastoma (GBM) samples. However, no difference on survival times was found when compared GBM cases with upregulated and downregulated *STMN1* (*Breslow* = 0.092, median survival time: 11 and 13 months, respectively). Functional assays knocking down *MELK* by siRNA in GBM cell line showed that gene and protein expression of both MELK and stathmin were diminished. On the other hand, when the same analysis was performed for *STMN1*, only stathmin gene and protein was silenced.

**Conclusions:**

The results presented herein point stahtmin as a downstream target in the MELK pathway that plays a role in malignant progression of astrocytomas.

**Electronic supplementary material:**

The online version of this article (doi:10.1186/s12953-016-0094-9) contains supplementary material, which is available to authorized users.

## Background

Eighty percent of primary central nervous system malignant tumours are gliomas, and astrocytomas constitute more than half of gliomas [1]. According to the 2007 World Health Organization (WHO), these tumours are classified into four grades: grade I or pilocytic astrocytomas (AGI), grade II or diffuse astrocytomas (AGII), grade III or anaplastic astrocytomas (AGIII) and grade IV (AGIV) or glioblastomas (GBM) [2]. GBM accounts for the majority of astrocytomas and can be further divided into two subgroups: primary GBM, which arises *de novo*, and secondary GBM, which results from the progression of a lower grade astrocytoma. Astrocytomas ranging from AGII to GBM are considered as diffusely infiltrating astrocytomas and therefore are not curable via resection due to the invasive malignant tumour cells in the surrounding normal brain tissue [2–5].

Furthermore, the therapeutic outcome of conventional treatments remains unsatisfactory [5, 6], and the overall 5-year survival rate of GBM remains less than 5 % and is even lower for elderly patients [7]. The identification of additional therapeutic targets remains a challenge for GBM treatment. In addition, novel molecules that are specifically mutated or overexpressed in cancer cells provide both mechanistic insight regarding tumour establishment and progression as well as potential targets for the development of new anticancer drugs. In this context, serine/threonine kinases represent an extensively examined protein class, and targeting these molecules in a specific manner has proven not only to be very effective but also to minimize unfavourable side effects [8].

In a previous report, our group has demonstrated a stepwise expression level increase of the serine/threonine kinase Maternal Embryonic Leucine Zipper Kinase (MELK) in parallel with the increasing degree of malignancy in astrocytomas [9]. Additionally, the relevance of *MELK* in other brain tumours, including medulloblastomas, meningiomas, and oligodendrogliomas, has been described [9, 10], along with many other solid tumour types, such as colorectal, lung, ovary [11], breast [12] and prostate tumours [13]. Moreover, MELK has been shown to be associated with poor prognosis in breast and prostate cancer patients [12, 13].

MELK is also known as murine protein serine/threonine kinase 38 (MPK38) [14] and Eg3 protein (pEg3) [15]. MELK is a cell cycle-dependent protein kinase that belongs to the KIN1/PAR-1/MARK family of serine-threonine kinases [15–17]. It is localised in the cytoplasm and nucleus during interphase and at the cell cortex during anaphase and telophase [18]. MELK, activated via autophosphorylation, phosphorylates several substrates which modulates intracellular signalling in several cellular and biological processes. Furthermore, MELK interacts by binding to the following molecules: a) cell cycle protein (CDC25B), inducing cell accumulation in G2 [16], b) zinc finger-like protein 9 (ZPR9), a physiological substrate of MELK kinase in vivo [19], c) mitosis-promoting factor (MPF) and mitogen-activated protein kinase (MAPK), enhancing kinase activity [20], d) members of the Bcl-2 family of proapoptotic genes (Bcl-GL), conferring resistance to apoptosis [21, 22], e) inhibitor of protein Ser/Thr phosphatase-1 (NIPP1), transcription and splicing factor, regulating cell cycle progression through pre-mRNA processing [23],f) PDK1, an enzyme responsible for Akt/PKB loop activation, inhibiting its activity and function, g) Smad proteins, Smad2, Smad3, Smad4, and Smad7, intracellular signalling mediators of the TGF-β signalling pathway, regulating their activity [24], and h) p53, tumour suppressor, enhancing p53-dependent apoptosis and arresting cycle cell by modulating p53 stability [25].

MELK is also involved in embryonic development [19, 26] and is enriched in multiple multipotent neural progenitor-containing populations. MELK induces expression of POU5F1, a well-known stem cell marker, particularly in hematopoietic stem cells [27, 28].

MELK has been considered a potential target for cancer therapy because of the interaction with multiple proteins at distinct stages of tumorigenesis [29, 30], and the first MELK-specific small molecular compound, OTSSP167, was developed [29]. However, the downstream signalling pathway of MELK in cancer cells is still not fully understood, and the putative function of MELK in astrocytomas remains unclear. The discovery of genes and/or proteins involved in the MELK pathway might contribute to the development of additional compounds to improve treatment effectiveness.

In the present study, we performed a comparative proteomic analysis and used *oligonucleotide microarrays* to analyze the differential pattern of expression before and after knocking down *MELK* to investigate the proteins and genes involved in the signalling pathways. Additionally, we have selected one MELK-associated protein to further investigate its function in astrocytoma progression. The protein stathmin and its coding gene (*STMN1*) presented lower expression when *MELK* was silenced.

## Results

### Stathmin is one of the proteins identified as down-regulated when MELK is knocked down

**Fig. 1:**
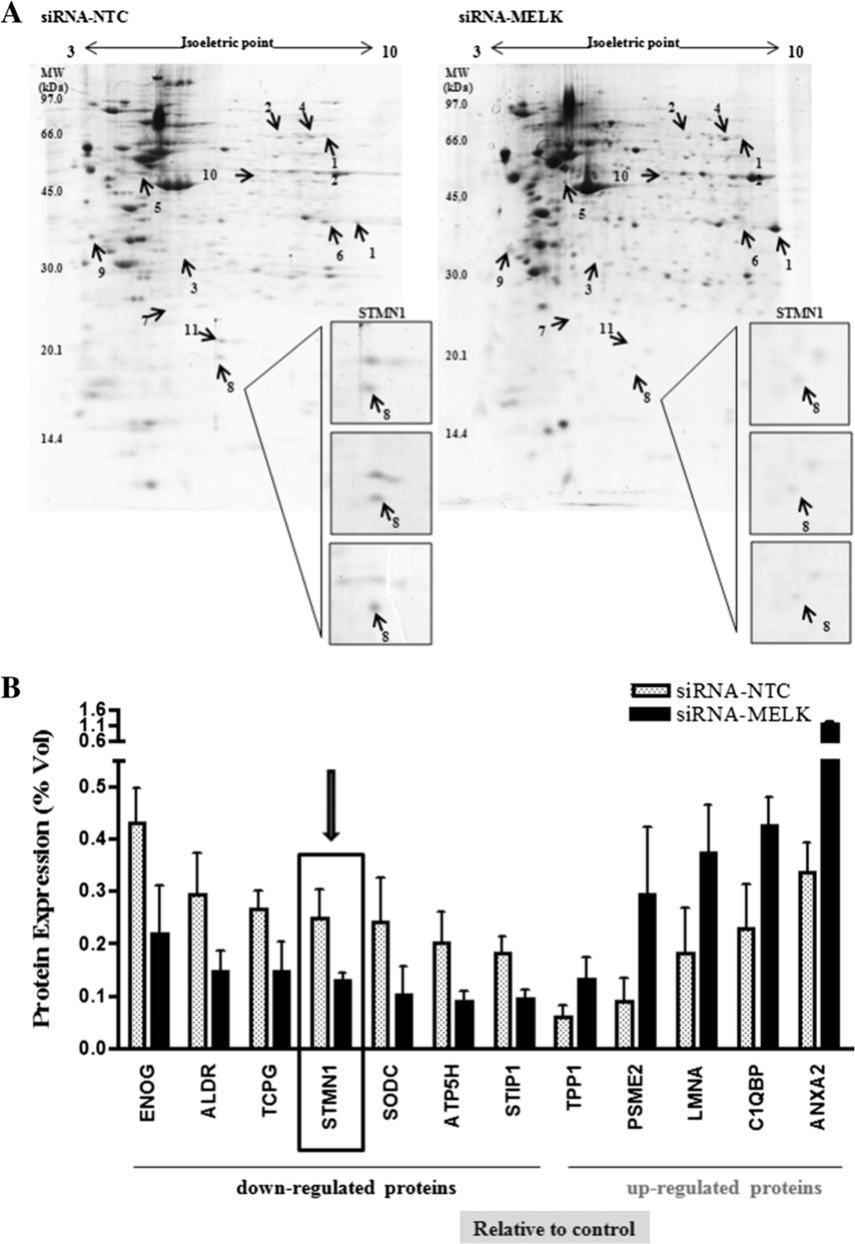
Proteomics analysis of differentially expressed proteins in U87MG transfected with siRNA for *MELK* compared with U87MG transfected with non-target control. **a** Representative two-dimensional electrophoresis gels (2DE). Protein extracts (200 μg) were applied to 2DE using IPG pH 3-10NL (7 cm) for isoelectric focusing and 12.5 % SDS-PAGE mini gels as a second dimension. Gel image analysis was performed using ImageMaster 2D Platinum v.7.0 software. The arrows indicate selected protein spots from mass spectrometry analysis. **b** Proteins differentially expressed (up-regulated and downregulated) in the glioma cell line U87MG transfected with siRNA for *MELK* compared with U87MG cells transfected with non-target control (NTC). The expression levels of 12 differentially expressed protein spots were quantified on the basis of the normalised volume of the 2DE spots (% vol) for each group. These data were analysed via ANOVA (p < 0.05). The data are reported as the mean ± SD. One of the proteins, Stathmin (STMN1), exhibited lower expression levels when *MELK* was silenced

A proteomic study based in 2DE was performed to analyze proteome changes in U87MG cells in which *MELK* was knocked down. The transfection reduced efficiently MELK expression at gene and protein levels. Twelve differentially expressed proteins (Table 1) were identified via 2D gel image analysis (Fig. 1a) and mass spectrometry. The differentially abundant proteins are shown in Fig. 1b, considering the % volume of the spot to be equivalent to the expression level (n = 3, mean ± SD, *p* < 0.05). Five proteins displayed up-regulation after *MELK* silencing: tripeptidyl-peptidase 1 (TPP1), proteasome activator complex subunit 2 (PSME2), lamin-A/C (LMNA), complement component 1 Q subcomponent-binding protein, mitochondrial (C1QBP) and annexin A2 (ANXA2). On the other hand, seven proteins presented down-regulation when *MELK* was knocked down: gamma-enolase (ENOG), aldose reductase (ALDR), T-complex protein 1 subunit gamma (TCPG), stathmin (STMN1), superoxide dismutase [Cu-Zn] (SODC), ATP synthase subunit d, mitochondrial (ATP5H) and stress-induced-phosphoprotein 1 (STIP1) as shown in Table 1, Fig. 1b, Additional file 1: Table S1.

**Table 1:** Protein identification by MALDI-TOF-TOF MS of tryptic peptides obtained from 2DE spots of siRNA-NTC- and siRNA-*MELK*-transfected cells

Protein spot Id	Acc UniProt	Protein identification	Protein fold change MELK/NTC (% Spot Vol)	Protein score	MW/pI	Protein matches	Coverage (%)
1	ANXA2_HUMAN	Annexin A2	3.49 ± 0.08	1003	38832/7.57	13	41.6
2	TCPG_HUMAN	T-complex protein 1 subunit gamma	0.55 ± 0.06	395	61104/6.1	9	19.4
3	PSME2_HUMAN	Proteasome activator complex subunit 2	3.24 ± 0.13	356	27532/5.44	5	28.5
4	STIP1_HUMAN	Stress-induced-phosphoprotein 1	0.52 ± 0.02	325	63266/6.4	6	13.8
5	ENOG_HUMAN	Gamma-enolase	0.51 ± 0.09	246	47610/4.91	4	11.1
6	ALDR_HUMAN	Aldose reductase	0.50 ± 0.04	101	36252/6.51	3	11.4
7	ATP5H_HUMAN	ATP synthase subunit d, mitochondrial	0.44 ± 0.02	152	18548/5.21	4	24.2
8	STMN1_HUMAN	Stathmin	0.52 ± 0.01	145	17302/5.76	2	16.8
9	C1QBP_HUMAN	Complement component 1 Q subcomponent-binding protein, mitochondrial	1.86 ± 0.06	141	31761/4.74	2	9.9
10	TPP1_HUMAN	Tripeptidyl-peptidase 1	2.21 ± 0.04	139	61761/6.01	4	10.7
11	SODC_HUMAN	Superoxide dismutase [Cu-Zn]	0.42 ± 0.06	104	16164/5.7	1	9.1
12	LMNA_HUMAN	Lamin-A/C	2.08 ± 0.09	83	74424/6.57	4	6

### *STMN1* was confirmed as down-regulated after knocking down *MELK*

Following the selection criteria regarding data mining, gene annotation and *in silico* validation from an initial 44,000 genes analyzed on the oligonucleotide microarray assays, we found 667 overrepresented common genes with a fold change (NTC/MELK) ≥ 2 in two independent experiments. After gene annotation based on a public database, we found 87 overrepresented genes via hypergeometric test (*p* < 0.05) and 26 genes functionally associated with tumorigenesis. Subsequently, we checked the expression of these genes *in silico* based on our previous microarray data [9]. Five genes out of 26 were selected as down-regulated in astrocytomas, preferentially in higher malignant grades and tumour-related functions. These genes were metallopeptidase domain 9 (*ADAM9*), EPH receptor B4 (*EPHB4*), lectin, galactoside-binding soluble 12 (*LGALS12*), WNT1 inducible signalling pathway protein 1 (*WISP1*) and stathmin (*STMN1*) as shown in Additional file 2: Figure S2, Additional file 3: Table S3 and Additional file 4: Figure S4).

Interestingly, *STMN1* was the common target that was down-regulated together with *MELK* as detected by proteomic and microarray analyzes. Therefore, *STMN1* expression was further analyzed simultaneously with *MELK* gene expression in a series of astrocytomas of different malignant grades and non-neoplastic (NN) brain tissues.

### *MELK* and *STMN1* expression levels are higher in the most malignant astrocytomas

**Fig. 2:**
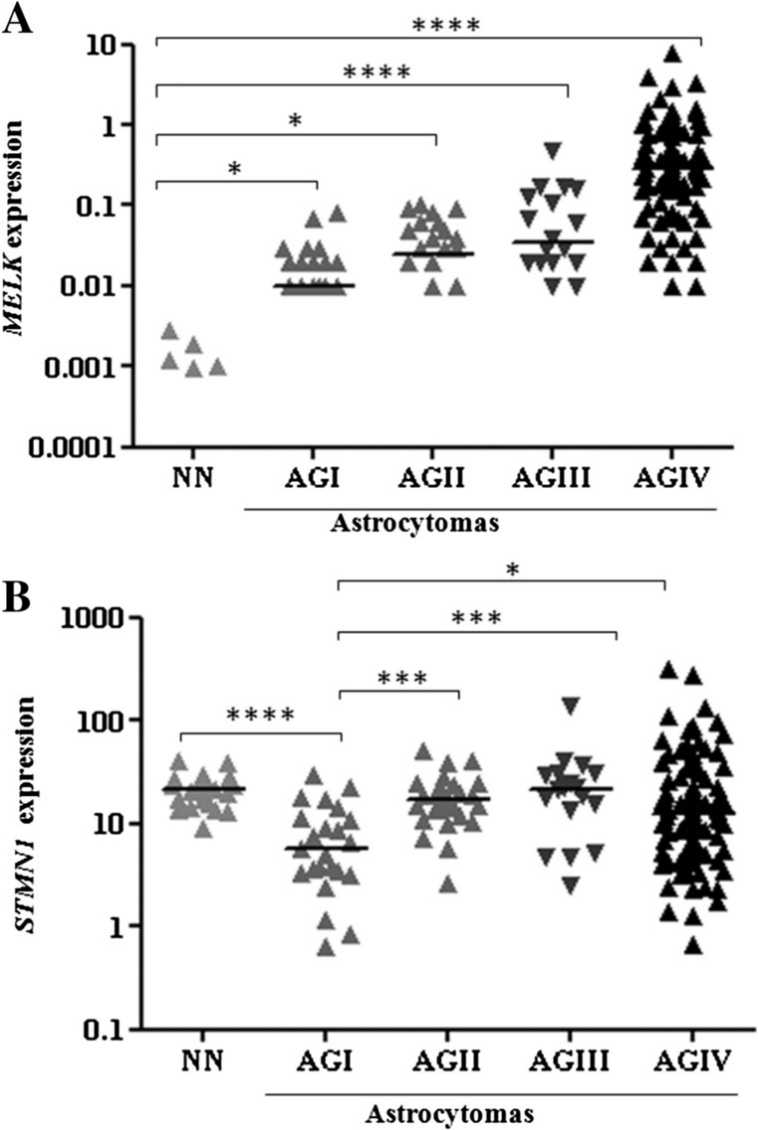
Gene expression levels in astrocytomas of different malignant grades (AGI, AGII, AGIII and GBM) and non-neoplastic brain tissues (NN). Expression levels of *MELK* **a** and *STMN1* (**b**) were analyzed by quantitative real-time PCR (qRT-PCR) using the SYBR Green method. The following equation was applied to calculate gene expression levels: 2^-∆Ct^, in which ∆Ct = Ct of the target gene-geometric mean Ct of reference genes. Horizontal bars indicate the median relative expression levels of all tissues. The differences in *MELK* and *STMN1* expression levels among the groups were statistically significant (Kruskal-Wallis test, p < 0.0001). *MELK* expression levels were higher in GBM samples (median value = 0.28) than the other groups. *STMN1* median expression levels for NN, AGI, AGII, AGIII and AGIV were 21.7, 5.7, 16.9, 21.8 and 12.2, respectively. A post-hoc Dunn’s test was used to calculate the differences in expression between NN and each astrocytoma group (**** *p* < 0.00005, *** *p* < 0.0005, ** *p* < 0.005 and * *p* < 0.05). The results are presented in log10 scale for better visualisation

We analyzed *MELK* and *STMN1* expression levels in the series of tissue samples, which included control NN and astrocytoma of all malignant grades. *MELK* expression levels were higher in GBM samples (median value = 0.28, SD ± 1.04) compared with lower grades astrocytomas. Of note, the majority of NN tissues (17 out of 22) displayed that *MELK* expression was below the detection level of the method (Fig. 2a). A significant difference in *STMN1* expression levels was also observed among the groups (*p* < 0.0001), and diffusely infiltrative astrocytomas (AGII to IV) presented higher expression levels than non-invasive pilocytic astrocytomas (AGI) (*p* < 0.0005, *p* < 0.0005 and *p* < 0.05, respectively). Furthermore, *STMN1* expression levels were higher in NN brain tissue than in AGI samples (*p* < 0.00005) (Fig. 2b).

**Fig. 3:**
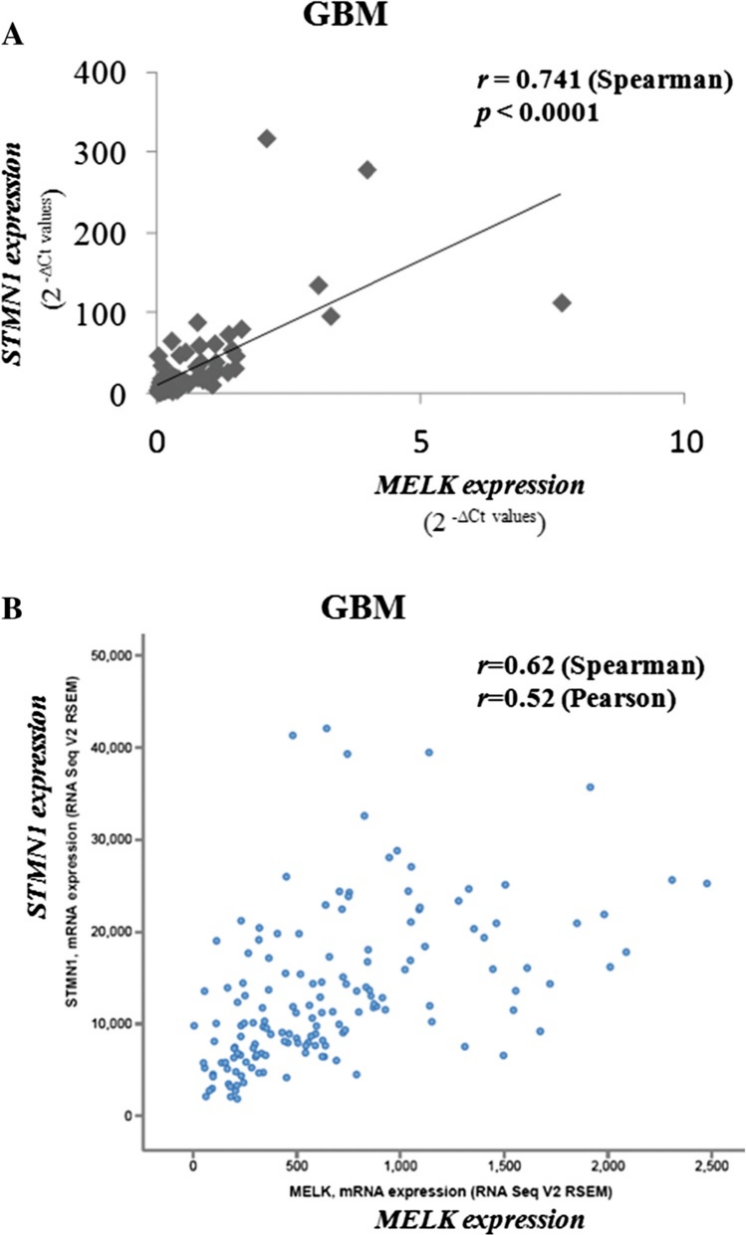
Correlation between *MELK* and *STMN1* gene expression levels in glioblastomas. qRT-PCR data analysis in our series of 86 cases **a** RNAseq data analysis in 154 cases TCGA was analyzed for co-expression of *MELK* and *STMN1* by z-Score (of RSEM) [54, 55] (**b**)

Moreover, *MELK* expression levels significantly correlated with *STMN1* expression levels in GBM samples (Fig. 3a). The correlation in gene expression also increased with the incremental increase in malignancy grade, as the association was non-significant in AGI (*r* =−0.008, *p* = 0.971), while it was slight in AGII (*r* = 0.330, *p* = 0.100), moderate in AGIII (*r* = 0.426, *p* = 0.088), and strong in GBM (*r* = 0.741, *p* < 0.0001) (Fig. 3a). These results of co-expression were confirmed in TCGA Research Network data of a GBM data set (*r* = 0.52 and 0.62, Person and Spearman correlation, respectively) as shown in Fig. 3b. Altogether, these findings support the hypothesis that *MELK* and *STMN1* may act in the same pathway, mainly in GBM.

### *MELK* and *STMN1* expression levels impact on overall survival of GBM patients

GBM cases were divided into increased expression and decreased expression status of *MELK* and *STMN1* according to their expression transcript levels.

However, no difference on survival times was found when compared GBM cases with upregulated and downregulated *STMN1* (*Breslow* = 0.092, median survival time at 11 and 13 months, respectively) and for *MELK* (*log rank*: 0.745)

### MELK and stathmin proteins are expressed in astrocytomas

**Fig. 4:**
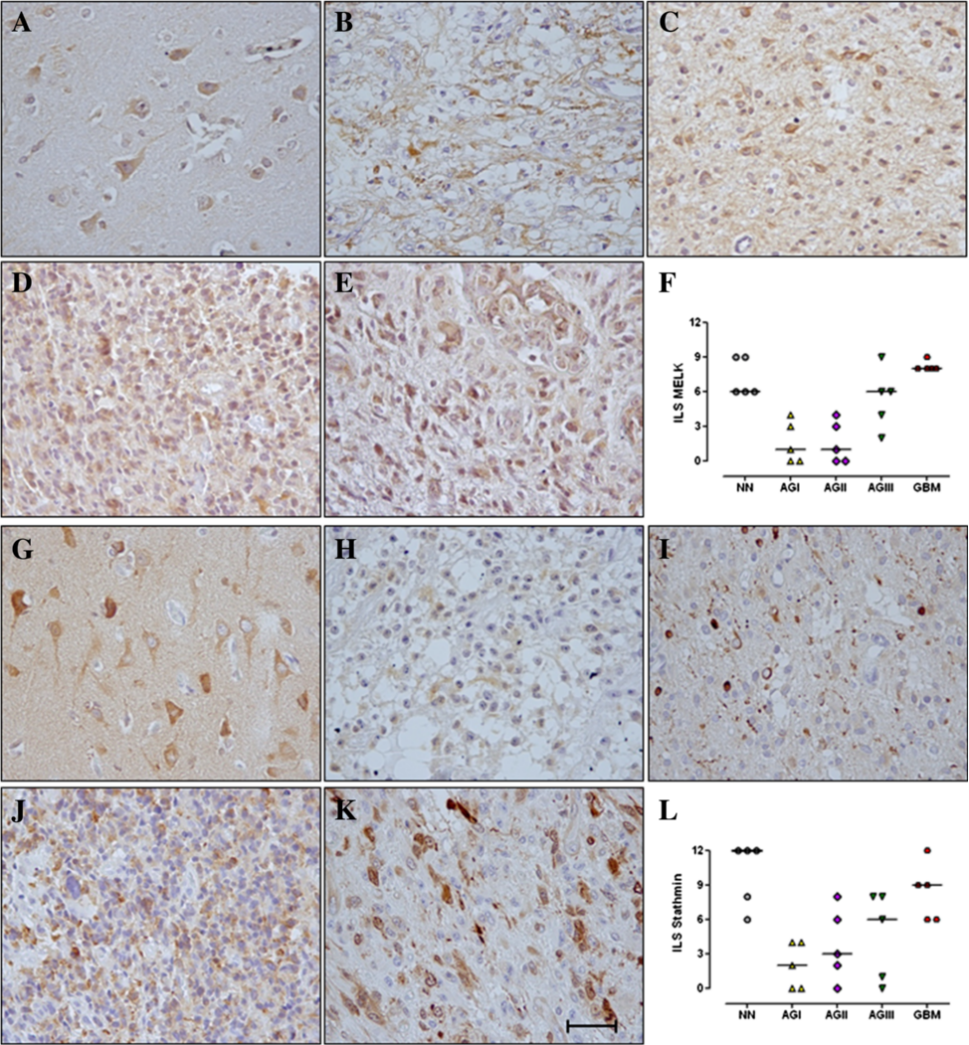
MELK and stathmin protein expression in non-neoplastic and astrocytoma tissue samples. Immunohistochemistry was performed for MELK **a**–**f** and stathmin (**g**–**l**) in 6 cases each of in (**a** and **g**) non-neoplastic (NN) brain tissues, (**b** and **h**) pilocytic (AGI), (**c** and **i**) low grade (AGII), (**d** and **j**) anaplastic (AGIII) astrocytomas, and (**e** and **k**) glioblastoma (GBM). Pictures show representative cases of each sample type, with 400-x magnification. The bar (**k**) represent the scale bar of 10 μm. The graphs (**f** and **l**) represent a semi-quantitative immunolabelling score (ILS) calculated as the product of staining intensity and the percentage of positive cells. Both MELK and stathmin showed positive staining in the cytosol. MELK reactivity increased with the increment of the malignancy among diffusely infiltrating astrocytomas (AGII-AGIV). Stathmin cytosol staining was stronger in GBM cases. The horizontal bars show the median ILS of each group

MELK and stathmin were both in the cytosolic localization. MELK and stathmin expressions were detected in in different levels of NN brain cells. Nevertheless, stathmin expression levels were higher than MELK expression levels in NN tissues (the cells with morphological characteristics of neurons) (Fig. 4a and g). Both MELK and stathmin expressions in astrocytoma were higher in more malignant astrocytomas (Fig. 4b–e and Fig. 4h–k, respectively), as represented in the semi-quantitative analysis (4 F and 4 L, respectively).

### Stathmin is downstream in the MELK pathway

**Fig. 5:**
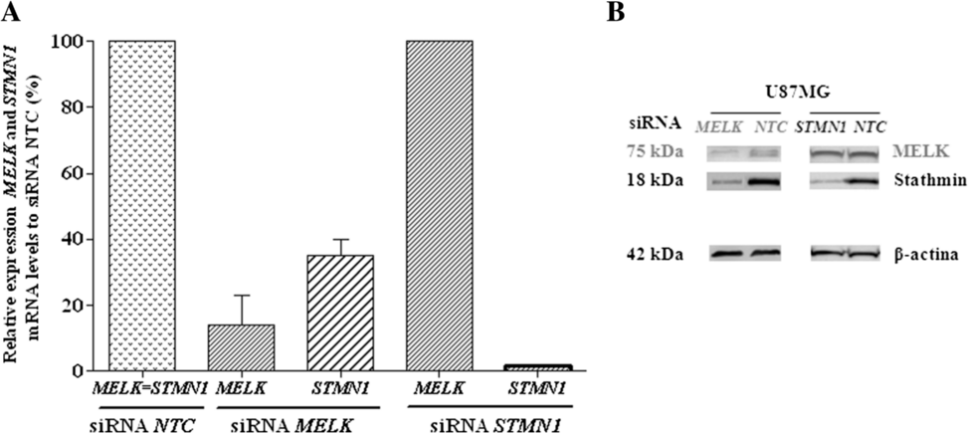
Effect of *MELK* and *STMN1* knockdown with siRNA in GBM cell line. *MELK* and *STMN1* expression levels relative to non-targeting control (NTC) were analyzed two days after transfection of U87MG cell line **a** The data show the average of two independent experiments and the vertical bar represents the standard deviations. Western blot of MELK and stathmin were analyzed after trasfection with siRNA for both genes and NTC (**b**)

Expression of *MELK* and *STMN1* was knocked out by specific siRNA transfection of U87MG glioma cell line. The gene and protein expression levels of both targets were checked in both conditions in two independent experiments. When *MELK* was efficiently silenced (95 and 70 % compared to NTC), *STMN1* expression was also considerably diminished to 70 % (Fig. 5a). In contrast, when *STMN1* was knocked out (99 and 98 % relative to NTC), no change in *MELK* expression was detected (Fig. 5b). Altogether, these data confirmed that stathmin is downstream MELK pathway.

## Discussion

In our previous study, we have also demonstrated that *MELK* knockdown in malignant astrocytoma cell lines caused a reduction in proliferation and anchorage-independent growth in in vitro assays [9]. The present study reveals stathmin as a novel target involved in the MELK pathway, based on both proteomic and gene expression approaches, using the strategy of *MELK* knock down with siRNA. We have previously shown a significant correlation between *MELK* expression and astrocytoma malignant grade, with the highest expression levels in GBM samples [9]. Herein, we observed that *MELK* expression also correlated with *STMN1* expression levels in GBM samples, both in our series and TCGA GBM samples.

Stathmin was the common target detected in both proteomic and oligonucleotide microarray analyzes, when comparing protein and gene profiles before and after *MELK* silencing. Furthermore, this target was selected in the present study because of its involvement in many important features of different types of tumors as invasion and metastasis, proliferation, survival, tumor grade and prognosis. Stathmin is a cytosolic phosphoprotein initially identified in neuroendocrine cells of the pituitary under conditions of stimulation with thyreotropin-releasing hormone (TRH) [31]. Stathmin regulates microtubule dynamics during cell cycle progression [32]. Non-phosphorylated or active stathmin promotes depolymerisation of microtubules by sequestering tubulin, while phosphorylated or inactive stathmin increases microtubule stabilisation and promotes mitotic spindle formation [33]. Although cells proceed through anaphase and telophase, fluctuations in microtubule polymerisation and depolymerisation lead to structural reorganisation and eventual spindle disassembly, followed by exit from mitosis and cytokinesis. Microtubules then reorganise into a new interphase cytoskeleton upon entry into a new cell cycle [34].

We detected higher STMN1 expression levels in diffusely infiltrative astrocytomas (AGII to AGIV) compared with AGI. Furthermore, *STMN1* expression levels were higher in NN brain tissue than in AGI samples. This is in concordance with the previously demonstrated function of stathmin in cell proliferation, as its level increases during the S and G2/M phases and decreases when cells accumulate in the G1 phase [35]. Stathmin also plays a role in neoangiogenesis, as high grade glioma-derived microvascular endothelial cells treated with *STMN1* siRNA reduce cell viability, increase apoptosis rates and suppress significantly the endothelial cell migration [35]. Moreover, stathmin phosphorylation at serine 16 mediates the ‘pioneering’ of microtubules into the leading edge of migrating cells, acting downstream Rac1 and Pak pathways [36]. Stathmin has been previously reported to act as a substrate of several intracellular signalling kinases, as mitogen-activated protein kinases (MAPKs) and phosphoinoinositide 3-kinase (PI3K) and phosphorylation at serine residues is the main mechanism of regulation of its activity [37]. Stathmin can be dephosphorylated by protein phosphatise 1 (PP1), protein phosphatase 2A (PP2A)and protein phosphatase 2B (PP2B) [38–40].

Therefore, stathmin plays a major role in tumour progression and dissemination rather than tumour initiation as suggested, independent of the histology of the primary tumour. The reduced expression of *STMN1* in AGI samples compared with NN brain tissue and a stepwise increase that parallels the incremental increase in malignant grade in higher grades of invasive astrocytomas, as shown in the present study, corroborate this suggestion.

**Fig. 6:**
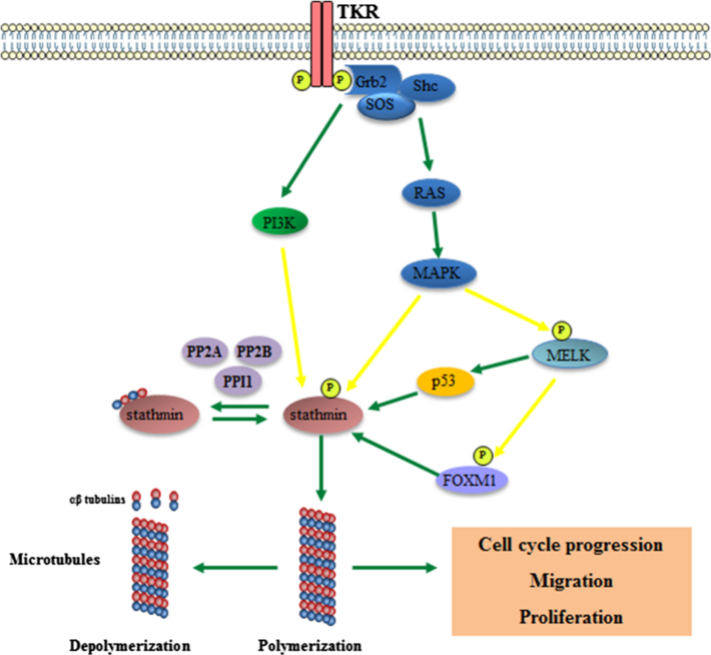
MELK and stathmin signaling. MELK can induce stathmin expression through two transcription factors, p53 and FOXM1. A tyrosine kinase receptor (TKR) is activated by phosphorylation after binding to a ligant. Growth factor receptor-bound protein 2 (GRB2) binds to the phophorylated residue of TKR and to Son of Sevenless homologs (SOS). GRB/SOS complex activates phosphoinositide 3-kinase (PI3K) and RAS-MAPK signaling pathways. Activation of mitogen-activated protein kinase (MAPK) and phosphoinositide 3-kinase (PI3K) signaling pathways results in phosphorylation of stathmin on serine sites and consequent microtubule stability and cell cycle progression. Stathmin may be dephosphorylated by protein phosphatase 1 (PP1), protein phosphatase 2A (PP2A) and protein phosphatase 2B (PP2B), resulting in microtubule instability. TKR: tyrosine kinase receptor; P: phosphorylation

The associated expression of *MELK* and *STMN1* demonstrated in GBM cases on this study and TCGA public data may be explained by two pathways, via p53 or FOXM1. FOXM1 is a master transcription factor that regulates mitotic progression of different types of cancer cells and forms a protein complex with MELK in glioma stem-like cells, leading to phosphorylation and activation of FOXM1 in a MELK kinase-dependent manner [41]. FOXM1 induces *STMN1* expression in B-cell lymphoma [42], breast cancer [43] and gastric cancer [44]. On the other hand, MELK carboxyl-terminal domain can physically interact with the central DNA-binding domain of p53, promoting p53 activity through direct phosphorylation at Ser15. Consequently, activated p53 promotes apoptosis and cell cycle arrest, as well as transcription of target genes, including *STMN1*, which has a promoter with p53-binding domain [45, 46]. Also, MELK both regulates and is regulated by one family of MAP kinases, the c-Jun NH(2)-terminal kinases (JNKs) in a cancer-specific manner [47]. The tumor-specific MELK interaction with the JNK/c-JUN is likely one of the mechanisms for the selective apoptosis that occurs as a result of MELK inhibition in GBM stem cells, but not in normal progenitors. These cells give rise to a variety of tumor cells in response to cellular and environmental signals, and may play a role in tumor initiation and propagation [48]. A proposal of the signaling pathway of MELK that highlights stathmin activation via FOXM1 and p53 is presented in Fig. 6, where a disturbance on the pathway involving MELK and stathmin may lead to alterations of tumor cell cycle progression, proliferation, and migration.

A *MELK*-specific small molecule compound, named OTSSP167, displayed significant suppression of phosphorylation levels of PSMA1 (proteasome subunit alpha type 1) and DBNL (drebrin-like). Both of these targets were identified as novel MELK substrates, and they proved to be important for stem cell characteristics and invasiveness. This compound suppresses mammosphere formation in breast cancer cells and has exhibited significant tumour growth suppression in xenograft studies using breast, lung, prostate, and pancreatic cancer cell lines in mice via both intravenous and oral administration [8]. Actually, MELK seems to be a druggable target as *MELK* overexpression has also been associated with poor prognosis in breast and prostate cancer [12, 13]. Nevertheless, in the present series of astrocytomas, no correlation was observed between GBM patient survival outcome and *MELK* and *STMN1* expression status. In contrast, *STMN1* overexpression has been associated with poor survival as well as with local or distant metastasis formation in several types of human cancer, including bladder, breast, cervical, colorectal, gastric, head and neck, hepatocellular, ovarian, prostate and urothelial carcinomas [37]. Therefore, these evidences suggest that combined targeting of MELK and stathmin might yield a better result. However, the high expression levels of stathmin in normal brain may be an impediment to its eligibility as a therapeutic target for brain tumours. Therefore, further search for other druggable targets downstream stathmin and MELK might help to establish other therapeutic strategies.

## Conclusions

The results presented herein point stathmin as a downstream target in the MELK pathway that plays a role in malignant progression of astrocytomas. The effective role of these downstream targets on the MELK-stathmin network in the cell cycle process may be explored in future studies. In addition, more detailed mechanisms with upstream and downstream signaling MELK pathways still need to be identified.

## Methods

### Cell culture and transient transfection with siRNA

The human malignant astrocytoma cell line U87MG was obtained from the American Type Culture Collection (Manassas, VA, USA), and the cells were cultured in Dulbecco’s modified Eagle’s medium (DMEM) supplemented with 10 % fetal calf serum (FCS), 100 IU/ml penicillin and 100 μg/ml streptomycin in a humidified incubator at 37 °C with a controlled 5 % CO_2_ atm.

A total of 1x10^5^ U87MG cells were seeded in a six-well plate and transfected after 24 h with *MELK* siRNA (GUAUAAAGCCAUUACAUUAUCAUCAUC) and *STMN1* siRNA (UACUAAGUGCUGUCCACUAAUAUGCAC) (27mers double-stranded RNA dicer-substrate, IDT, Coralville, IA) and cells were also transfected with using Lipofectamine RNAiMAX (Life Technologies, Carlsbad, CA). Control cells were transfected with scrambled non-targeting control (NTC) siRNA (IDT). siRNA concentrations for *MELK* and *STMN1* were 10 nM and 0.1nM, respectively. *MELK* and *STMN1* knocking down was assessed using quantitative real time PCR (qRT-PCR) and Western blot after 48 h of transfection. For functional analysis of *MELK* and *STMN1* knockdown after siRNA transfection, the *HPRT* was used as a reference gene. The expression values were calculated relative to the control *NTC*.

Experiments were performed in duplicates (gene expression analysis) and triplicates (proteomic analysis).

We analysed if MELK is upstream or downstream of STMN1 in the pathway, then we tested the expression of MELK and STMN1 using qRT-PCR and Western blotting in two conditions: before and after U87MG cell line was transfected (1x10^5^ cells) with: a) 10nM siRNA-*MELK* and 10nM siRNA-*NTC* and b) 0.1nM-siRNA-*STMN1* and 0.1nM siRNA-*NTC*

### Proteomics

#### Protein extraction

Cell pellets of siRNA-NTC and siRNA-*MELK* U87MG cells were resuspended in lysis solution with 7.7 M urea, 2.2 M thiourea, 4 % CHAPS and a protease inhibitor cocktail (Sigma Aldrich, St. Louis, MO). The cell pellets were then placed in an ultrasound bath (UltraSonic Clear 750, UNIQUE) for three cycles of 5 min for complete pellet resuspension and subsequently centrifuged at 20,000xg for 30 min at 4 °C. Protein concentration was determined using the Bradford protein assay method.

#### 2DE and image analysis

For the first dimension, Immobiline Dry IPG strips (7 cm, pH 3–10 non-linear; GE Healthcare, Uppsala, Sweden) were re-hydrated for 12 h with 200 μg of each sample, 0.5 % IPG buffer 3–10 non-linear, 0.3 % DTT and electrophoresis lysis buffer until reaching the final volume of 125 μL. The isoelectric focusing was performed in an IPGphor system (Ettan IPGphor III, GE Healthcare) at 20 °C with a constant current of 50 mA per IPG strip until an accumulation of 40,000 Vh was reached. After isoelectric focusing, the IPG strips were reduced with DTT and alkylated with iodoacetamide, and SDS-PAGE 12.5 % was then performed as the second dimension. The proteins were stained with colloidal Coomassie blue. The gel images were acquired with a transmissive scanner (ImageScanner, Pharmacia-Biotech, GE Healthcare) using MagicScan software (GE Healthcare) and analyzed with Image-Master 2D Platinum v.7.0 software (GE Healthcare). The spot volume was measure and reported as percent volume of the spot (normalized volume of spots, % vol) in relation to the sum of all detected spot and this provided normalized spot volumes. Differential protein abundance was detected on the basis of relative % vol. Reproducibility was determined by the number of matched spots in relation to the reference group (control). Differential protein levels were detected on the basis of a relative % volume ratio for each group, and the protein spots were accepted as differentially expressed when *p* < 0.05 (ANOVA).

#### Trypsin digestion and mass spectrometry analysis

Selected protein spots were manually excised from the gels. SDS and Coomassie blue were removed by successive washes of 50 % acetonitrile in 0.1 M of ammonium bicarbonate pH 7.8, followed by dehydration in neat acetonitrile and drying in a Speed Vac (Savant, New York, NY). Proteins were then digested with 0.5 μg modified trypsin (Promega Corp., Madison, WI) in 0.1 M ammonium bicarbonate for 18 h at 37 °C. The reaction was stopped by adding 1 μL of neat formic acid. Tryptic peptides were extracted via passive elution and desalted in micro-tips filled with reverse phase resin (POROS R2, Perseptive Biosystems, Foster City, CA), which were previously equilibrated in 0.2 % formic acid. Samples were de-salted via two washes of 0.2 % formic acid (150 μL). Peptides were eluted in 30 μL of a 60 % methanol/5 % formic acid solution, concentrated in Speed Vac and resuspended in an α-cyano-4-hidroxicinamic acid matrix solution (5 mg/mL). Two to five microliters of each sample was loaded into the MALDI target and analyzed via MALDI-TOF-TOF mass spectrometer (Axima Performance—Kratos-Shimadzu, Manchester, UK).

Two to five microliters of each sample was loaded into the MALDI target and analyzed by MALDI-TOFTOF mass spectrometer (Axima Performance—Kratos-Shimadzu, Manchester, UK). MALDI-TOF/TOF was calibrated with a synthetic peptide mixture of Bradykinin fragment 1–7 (757.40); Angiotensin II (1046.54); P14R (1533.85) and ACTH fragment 18–39 (2465.20) (Sigma-Aldrich, Saint Louis, CA) in CHCA matrix. Mass spectra resolution were FWHM of 10,000 and accuracy <50 ppm which were collected for MS using 10 shots/profile with 64 profiles/spectrum at laser power of 90. CID-MS/MS spectra were selected as data dependent acquisition (DDA) for the 10th most abundant ions in MS spectra using 20 keV and helium as collision gas. Laser power was setting to 110 and 10 shots/profile with 256 profiles/spectrum. Mass spectra peak list was generated by Launchpad v. 2.8.2 software (Kratos-Shimadzu, Manchester, UK) as mascot generic format and only CID-MS/MS spectra were used for protein identification using home-licensed MASCOT server software (v 2.4.04).

#### Protein identification

PMF and CID-MS/MS spectra from 2 DE selected protein spots were submitted for protein identification by searching against the Swiss-Prot database version 57.2 and filtered for Homo sapiens (total of 20,402 human sequences) using MASCOT version 2.2.04. The database search parameters were set as follows: hydrolysis of trypsin, one missing cleavage was allowed, fixed modification for carbamidomethyl-Cys and variable modification for methionine oxidation. The mass tolerance for precursor ions was 1.2 Da, and the mass tolerance for fragment ions was set to 0.8 Da. Protein identification was supported by MS/MS analysis of individual ions by CID-MS/MS. Proteins were identified on the basis of at least two unique peptides if the score was higher than 35 (*p* < 0.05) or the amino acid sequence was consistently covered by a series of ion fragment b and y types.

### Microarray hybridisation and gene expression analysis

#### Total RNA extraction and cDNA synthesis

The total RNA was extracted from cell lines using RNeasy Mini Kit (Qiagen, Hilden, Germany). Synthesis of cDNA was performed via reverse transcription using oligo(dT), random hexamers, and SuperScriptIII (Life Technologies) according to the manufacturer’s recommendations.

#### Microarray hybridisation, gene expression analysis and Criteria of microarray analysis

Initially we performed a microarray analysis to select genes involved in *MELK* pathway. The differential gene expression profiles in the U87MG cell line transfected with MELKsiRNA compared with NTC-transfected cells was analyzed by 44 K DNA microarrrays (Whole Human Genome Microarray Kit, Agilent Technologies, Santa Clara, CA).

The human U87MG glioma cell lines transfected (1x10^5^ cells) with oligonucleotide MELK siRNA reduced MELK expression levels by 95 and 96 % in first and second independent experiments, respectively, at the 48 h confirmed by qRT-PCR and western blotting.

Hybridisation was performed according to the protocol provided by the manufacturer (One-Color Microarray-Based Gene Expression Analysis—Quick Amp Labeling, Agilent Technologies). The images were captured by the reader Agilent Bundle according to the parameters recommended for bioarrays and extracted using Agilent Feature Extraction software version 9.5.3, considering spots with none or only one flag. The selected transcripts were analyzed with the R software version 2.11.0 (R Development Core Team, 2008) and the Lowess test was applied for array normalisation. We obtained common genes with reduced expression when *MELK* was silenced compared with the control considering a fold change (NTC/MELK) ≥2 in two independent assays. These genes were annotated using WebGestalt [49].

### Workflow for gene selection process of involved in *MELK* pathway

#### Microarray 1

We considered one or more genes in each group as statistically significant at *p* < 0.05 by the hypergeometric test. Detailed analyzes of each up-regulation gene were performed using the Gene database of the National Center for Biotechnology Information (http://www.ncbi.nlm.nih.gov/gene) and the SAGE database of the Cancer Genome Anatomy Project (http://cgap.nci.nih.gov/Genes/GeneFinder).

#### Microarray 2

Selected differentially expressed genes were validated *in silico* with the astrocytoma microarray data (10 k DNA CodeLink Bioarrays-Human Uniset I; GE Healthcare,) (4 of pilocytic astrocytomas, 6 of low grade astrocytomas, 3 of GBMs and 2 pools of 3 non-neoplastic brain tissues) previously published [9]. We refined the selection by choosing down-regulation genes predominantly in GBM cases, preferentially in higher malignant grades and tumour-associated functions. The workflow for selection process of genes involved in *MELK* pathway was presented as Additional file 2: Figure S2).

## Tumour samples

One hundred and fifty-two astrocytomas (grades I to IV) and 22 non-neoplastic (NN) brain anonymized tissues from epilepsy patients subjected to temporal lobectomy were obtained during therapeutic surgery from patients treated by the Neurosurgery Group of the Department of Neurology at Hospital das Clinicas at the School of Medicine of the University of São Paulo. The cases were categorised according to the WHO grading system [2] by neuropathologist from the Division of Pathological Anatomy at the same institution. The studied series consisted of 22 AGI (mean age: 19.4 years), 26 AGII (mean age: 34 years), 18 AGIII (mean age: 35 years), 86 GBM (mean age: 54 years), and 22 NN brain specimens (mean age: 38 years), according to demographic data presented in our previous study [50]. Samples were macrodissected and immediately snap-frozen in liquid nitrogen upon surgical removal. Necrotic, cellular debris and non-neoplastic areas were removed by microdissection from the frozen block prior to RNA extraction [9, 51]. Written informed consent was obtained from all adult patients in accordance with ethical guidelines, and this project was approved by the local ethical committee. This study was approved by the Ethic Committee of School of Medicine of the University of São Paulo (Protocol# 0263/07).

### Total RNA extraction, reverse transcription and qRT-PCR

The total RNA was extracted from frozen tissues using an RNeasy Mini Kit (Qiagen). The first strand of cDNA was synthesised from 1 μg of total RNA previously treated with 1 unit of DNase I (FPLC-pure, GE Healthcare) using random hexamers, oligo(dT) primers, RNase inhibitor, and SuperScript III reverse transcriptase according to the manufacturer’s recommendations (Life Technologies). The resulting cDNA was subsequently treated with one unit of RNaseH (GE Healthcare), diluted with TE buffer, and stored at−20 °C until later use.

The relative expression levels of *MELK* and *STMN1* were analyzed by qRT-PCR using the SYBR Green approach. Quantitative data were normalised using the geometric mean of three reference genes suitable for the analysis: hypoxanthine phosphoribosyltransferase (*HPRT*), glucuronidase beta (*GUSB*) and TATA box-binding protein (*TBP*), as previously demonstrated by our group [52]. The primers were synthesised by IDT as follows (5′ to 3′): *MELK* F: AAACCCAAGGGTAACAAGGA, *MELK* R: ACAGTATGCCCATGCTCCAA, *STMN1* F: TGTCGCTTGTCTTCTATTCACCAT, *STMN1* R: CTTTTGACCGAGGGCTGAGA, The primer concentrations used were 200 nM for *HPRT*, *TBP*, and *STMN1* and 400 nM for *MELK* and *GUSB*. SYBR Green I amplifications were conducted on an ABI Prism 7500 sequence detector (Applied Biosystems, Foster City, CA) with an incubation for 2 min at 50°, 10 min at 95°, followed by 40 cycles of 15 s at 95° and 1 min at 60 °C. All reactions were performed in duplicates and the following equations were applied to calculate gene expression levels: 2^−ΔCt^, in which ΔCt = Ct target gene-geometric mean Ct of reference genes [53]. For cellular gene expression analysis, only *HPRT* was used as reference gene, and relative expression was calculated using NTC as reference sample.

### Analysis of TCGA GBM gene expression data set

*MELK* and *STMN1* gene expression levels were analysed in the cBio Portal for Cancer Genomics database (http://www.cbioportal.org) [54]. RNAseq data set of 154 cases of GBM [55] was analyzed for co-expression of *MELK* and *STMN1* by z-Score (of RSEM).

### Western blot analysis

After transfection with control siRNA (*NTC*) and siRNA specific for *MELK* and *STMN1*, total protein lysates were prepared from cell cultures with RIPA lysis buffer and protease inhibitor cocktail (Sigma-Aldrich) on ice. The protein concentration was determined using the Bradford reagent (Bio-Rad Laboratories, Richmond, CA) in duplicates with a standard curve of bovine serum albumine. The absorbance was measured at 595 nm using a microplate spectrophotometer (Multiskan Spectrum; Thermo Labsystems, Helsinki, Finland). Total protein lysates (30 μg) were separated by 12 % SDS polyacrylamide gel electrophoresis (TGX Mini Protean, Bio-Rad) with Tris-glycine running buffer and transferred to a nitrocellulose membrane using the iBlot dry blotting system (Life Technologies). The membrane was blocked with 5 % skim milk and incubated with primary polyclonal rabbit anti-MELK antibody (1:2000, Sigma-Aldrich) and primary monoclonal rabbit anti-Stathmin antibody (1:1000, Spring Bioscience, clone SP49, Pleasanton, CA). The membrane was also incubated with mouse monoclonal anti-β-actin (1:5000, clone AC-74, Sigma-Aldrich) as a protein loading control. The secondary antibodies used were anti-rabbit (1:2000) and anti-mouse IgG (1:5000) conjugated to peroxidase (Sigma-Aldrich). The immune complexes were visualized using enhanced chemiluminescence reagent (Western Lightning Chemiluminescence Reagent Plus, Perkin Elmer, Waltham, MA) and detected with ImageQuant LAS4000 (GE Healthcare).

### Immunohistochemistry analysis of Astrocytoma samples

We analyzed 20 astrocytoma cases (5 cases of each malignant grade) and 5 NN brain tissues. Serial 5-μm paraffin-embedded tissue sections of each case were deparaffinised, rehydrated and treated with endogenous peroxidase blocking reagent. After antigen retrieval with citric acid (10 mM pH 6.0) (Merck, USA) at 122 °C for 3 min using an electric pressure cooker (BioCare Medical, Walnut Creek, CA), sections were incubated with primary polyclonal rabbit anti-MELK, (1:750, Sigma-Aldrich) and primary monoclonal rabbit anti-Stathmin antibody (1:100, Spring Bioscience, clone SP49). The positive controls were normal testis and breast carcinoma samples for MELK and stathmin, respectively. The reactions were carried out using a commercial kit (Novolink, Novocastra, New Castle-upon-Tyne, UK) at room temperature with diaminobenzidine and Harris hematoxylin for nuclear staining.

MELK and stathmin expression levels were analyzed according to a semi-quantitative score system with both the intensity of staining and percentage of cells applied as follows: for intensity of staining, 0: negative, 1: weak, 2: moderate and 3: strong and for percentage of cells stained, 0: no cells stained, 1: 10–25 %, 2: 26–50 %, 3: 51–75 % and 4: 76–100 %. A immunolabelling score (ILS) was obtained by the product of the intensity of staining and the percentage of stained cells. The immunopreparations were analyzed by two independent investigators, and simultaneous revision were performed to obtain the final score in case the concordance was not achieved. Digital photomicrographs of representative fields were captured and processed using PICASA 3 (Google, Mountain View, USA).

### Statistical analyzes

Statistical normality test was performed using the Kolmogorov-Smirnov and then non-parametric Kruskal-Wallis and post-hoc Dunn’s tests were performed for analysis of the gene expression levels in all grades of astrocytoma and NN samples. Correlation between gene expression values in different groups of tumours was assessed using the Spearman-rho correlation tests (non-parametric test).

*MELK* and *STMN1* expression status was scored according to the median relative expression values (2^−ΔCt^) of each grade of astrocytoma. For statistical analysis, scores ≥ median values were defined as up-regulation and scores < median values as down-regulation.

Overall survival (OS) time was calculated as the interval between surgery and day of death in months. The *log-rank* test was used for univariate analysis to estimate differences in survival time compared with gene expression status (up- or down-regulation) in GBM cases according to Kaplan-Meier method; the relapse cases (n = 5) were excluded for this analysis. We considered *MELK* and *STMN1* gene expression status according to the median relative expression values of GBM (scores ≥ median values were defined as increased expression and scores < median values as decreased expression for both genes *MELK* and *STMN1*.The definition of *MELK* and *STMN1* mRNA expression status was scored according to the median expression values (2^−∆Ct^) of each grade of astrocytoma. A multivariate analysis was performed using the Cox proportional hazards model. The logistic regression model included the following parameters: age at diagnosis and gender. Differences were considered statistically significant when *p* < 0.05. The calculations were performed using SPSS for Windows, version 15.0 (Chicago, IL).

## Additional files

Additional file 1: Table S1. Table of differentially expressed proteins in U87MG transfected with siRNA for MELK compared with U87MG transfected with non-target control analyzed by proteomics. (XLS 79 kb) Additional file 2: Figure S2. Gene selection process of involved in *MELK* pathway. (TIF 3215 kb) Additional file 3: Table S3. Table of final selected genes by microarray analyzes. (XLS 22 kb) Additional file 4: Figure S4. Expression of final selected genes by microarray analyzes. (TIF 1753 kb)

## Abbreviations

MELK: Maternal Embryonic Leucine Zipper Kinase
qRT-PCR: quantitative real time PCR
STMN1: stathmin 1
siRNA: small interference RNA
NTC: non-target control
GBM: glioblastoma
