# Supplementary figures and images for: Stathmin involvement in the maternal embryonic leucine zipper kinase pathway in glioblastoma
Source: Proteome Sci. 2016 Mar 11;14:6. doi: 10.1186/s12953-016-0094-9 (PMC4788929; doi:10.1186/s12953-016-0094-9)

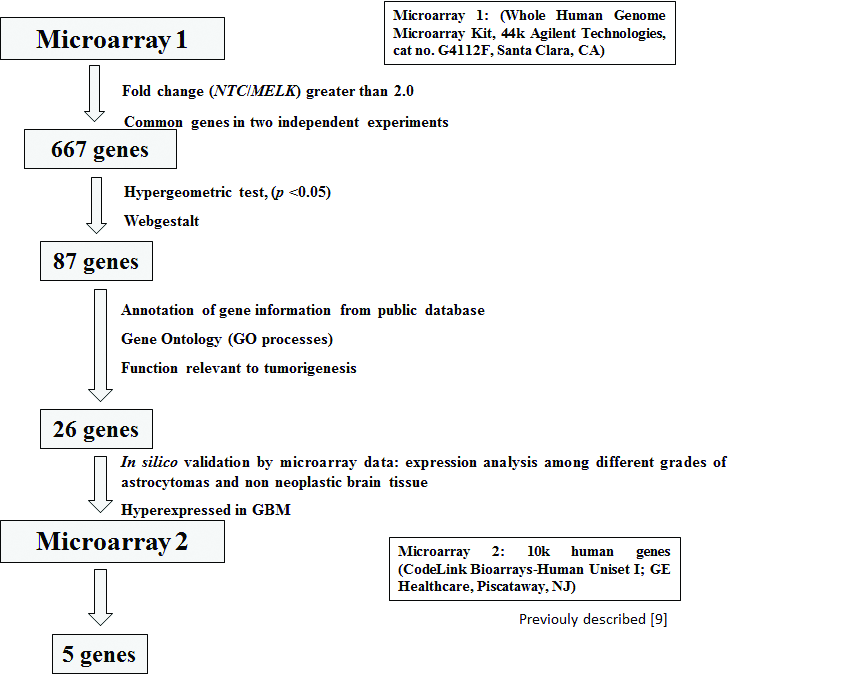

Supplement: Additional file 2: Figure S2. — Gene selection process of involved in MELK pathway. (TIF 3215 kb) [file 12953_2016_94_MOESM2_ESM.tif]

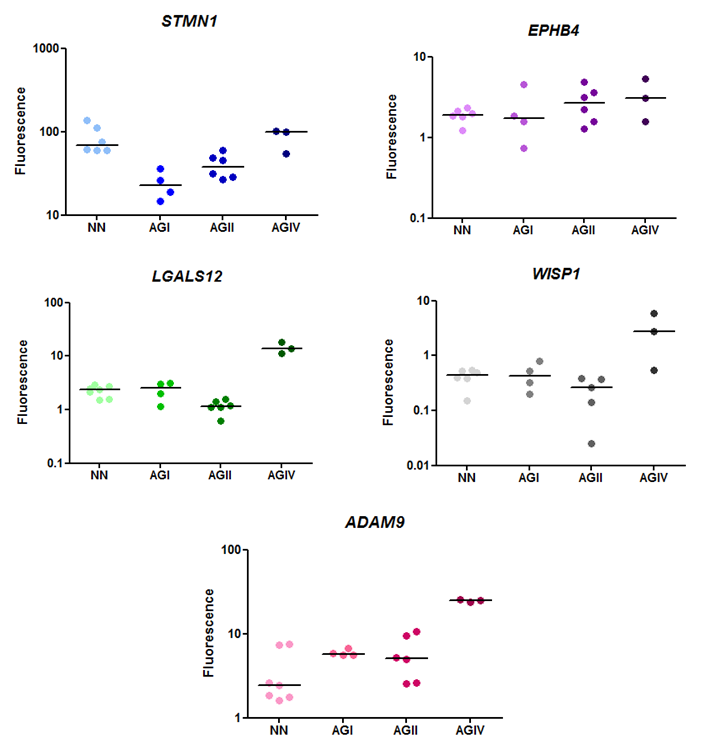

Supplement: Additional file 4: Figure S4. — Expression of final selected genes by microarray analyzes. (TIF 1753 kb) [file 12953_2016_94_MOESM4_ESM.tif]
